# A pragmatic randomized prospective trial of cooled radiofrequency ablation of the medial branch nerves versus facet joint injection of corticosteroid for the treatment of lumbar facet syndrome: 12 month outcomes

**DOI:** 10.1093/pm/pnad107

**Published:** 2023-08-14

**Authors:** Zachary L McCormick, Aaron Conger, Richard Kendall, Graham Wagner, A Michael Henrie, Madelaine Littell, Beau P Sperry, Russel Petersen, Amanda N Cooper, Masaru Teramoto, Taylor R Burnham

**Affiliations:** Department of Physical Medicine and Rehabilitation, University of Utah School of Medicine, Salt Lake City, UT 84108, United States; Department of Physical Medicine and Rehabilitation, University of Utah School of Medicine, Salt Lake City, UT 84108, United States; Department of Physical Medicine and Rehabilitation, University of Utah School of Medicine, Salt Lake City, UT 84108, United States; Department of Physical Medicine and Rehabilitation, University of Utah School of Medicine, Salt Lake City, UT 84108, United States; Department of Physical Medicine and Rehabilitation, University of Utah School of Medicine, Salt Lake City, UT 84108, United States; Department of Physical Medicine and Rehabilitation, University of Utah School of Medicine, Salt Lake City, UT 84108, United States; University of California Los Angeles School of Medicine, Los Angeles, CA 90095, United States; Alabama College of Osteopathic Medicine, Dothan, AL, United States; Department of Physical Medicine and Rehabilitation, University of Utah School of Medicine, Salt Lake City, UT 84108, United States; Department of Physical Medicine and Rehabilitation, University of Utah School of Medicine, Salt Lake City, UT 84108, United States; Department of Physical Medicine and Rehabilitation, University of Utah School of Medicine, Salt Lake City, UT 84108, United States

**Keywords:** low back pain, lumbar, facet joint, medial branch, ablation, steroids

## Abstract

**Background:**

Lumbar medial branch radiofrequency ablation (LRFA) and intraarticular facet steroid injections (FJI) are commonly performed for recalcitrant facet joint-mediated pain. However, no study has compared clinical outcomes of the two treatments in patients selected using dual medial branch blocks (MBBs) with an 80% relief threshold.

**Objective:**

Compare the effectiveness of cooled LRFA (C-LRFA) to FIJ as assessed by pain and functional improvements.

**Design:**

Prospective randomized comparative trial.

**Methods:**

Patients with dual MBB-confirmed facet joint-mediated pain were randomized to receive C-LRFA or FIJ. Outcomes were assessed at 1, 3, 6, and 12 months. The primary outcome was ≥50% improvement in numerical pain rating scale (NPRS) score at 3 months. Secondary outcomes included ≥30% Oswestry Disability Index (ODI) improvement and Patient Global Impression of Chance (PGIC) ≥6 points, among others. Data were analyzed using contingency tables and mixed-effects logistic regression models.

**Results:**

Of 1128 patients screened, 32 met eligibility criteria, were randomized, and received their allocated study treatment. In total, 20 (62.5%) and 12 (37.5%) participants received C-LRFA and FIJ, respectively. In the C-LRFA group, 70% (95% CI 48–85), 55% (95% CI 34–74), and 45% (95% CI 26–66) of participants met the NPRS responder definition, compared to 25% (95%CI 9–53), 25% (95% CI 9–53), and 17% (95% CI 5–45) in the FJI group at 3, 6, and 12 months, respectively (*P *=* *.014 at 3 months). The PGIC responder proportion was higher in the C-LRFA compared to FJI group at 3 and 6 months (*P *<* *.05).

**Conclusions:**

C-LRFA demonstrated superior success rates compared to FJI across pain and functional outcome domains.

**Trial registration details:**

ClinicalTrials.gov (NCT03614793); August 3, 2018.

## Introduction

Chronic low back pain (CLBP) is a common condition that contributes to substantial disability and healthcare cost in the United States.^1^^,^^2^ The lumbar facet joint has been implicated as an important source of CLBP in a subset of individuals, with prevalence estimates ranging from 15% to 45%.^3–7^ Elements of clinical history, physical examination, and imaging (radiographs, standard CT scan, standard magnetic resonance imaging [MRI] sequences) provide poor diagnostic specificity for pain of lumbar facet joint origin.^8^^,^^9^ Thus, clinicians have traditionally relied upon medial branch nerve blocks (MBBs) to confirm this diagnosis. The reference standard for the diagnosis of lumbar facet joint pain is a positive response to dual comparative MBBs with pain reduction of ≥80% of concordant duration to that expected of two different local anesthetics used on independent occasions.^10^^,^^11^ Furthermore, dual comparative MBBs have a high positive predictive value for determining the clinical outcome of lumbar medial branch radiofrequency ablation (LRFA), a commonly performed procedure for facet-mediated CLBP.^12^^,^^13^ Excellent clinical outcomes are reported in studies where patients are selected using these criteria and rigorous LRFA technique is implemented according to practice guidelines.^14^

In addition to LRFA, lumbar facet joint injection of corticosteroid (FJI) has historically been used as a treatment strategy for lumbar facet joint pain. Previous clinical outcome studies of FJI have shown variable results ranging from lack of superiority relative to intramuscular steroid injection to a 61% responder rate at 3-month follow-up.^15–18^ However, these studies may not accurately reflect the effectiveness of FJI because they have not utilized dual comparative medial branch blocks to confirm active lumbar facet joint pain.^10^ Alternatively, Kennedy et al. did study the effect of intraarticular facet joint steroid injection in a group of patients selected based on dual comparative branch blocks with an 80% relief threshold.^19^ The authors assessed time to LRFA and found that there was no intergroup difference, indirectly indicating that lumbar facet joint injection of steroid is no more effective than saline/sham. However, this study did not assess long-term pain or functional outcomes following FJI or LRFA.

Numerous studies^20–22^ have evaluated FJI and LRFA separately, but none have compared these two treatments head-to-head in a strictly selected population. Lakemeier compared FJI to LRFA but used a generous selection protocol^23^ consisting of a single positive MBB, which required only 50% relief in pain and not of concordant duration with that expected by the local anesthetic used.^23^ This block paradigm is known to produce a false positive rate of approximately 40%.^11^ Aditionally, a single LRFA lesion was applied with a 20 g conventional LRFA electrode; however, the publication contained no fluoroscopic images or additional procedural details confirming the parallel electrode technique necessary for conventional LRFA. The flawed selection criteria and ambigious LRFA technique utilized are similar to those used in the MINT Trials, which numerous experts in interventional pain, spine and radiology speciality societies have commented on and cited as evidence for the need to improve standards in research and clinical care.^24–27^ Given the considerable methodological limitations in the existing literature, research on clinical outcomes from studies adhering to more rigorous patient selection paradigms is warranted to compare the effectiveness of LRFA vs FJI, which led to the conception of the present study.

While the conventional radiofrequency ablation (RFA) modality has been studied extensively for LRFA, outcome literature on the effectiveness of cooled-tip (C-LRFA) technology is less developed. Outcomes studies addressing C-LRFA have been published, but no study has utilized the reference standard MBB paradigm described above or reported outcomes beyond 6 months.^28–30^ The spherical geometry and forward projection of the C-LRFA lesions beyond the distal end of the electrode allows the RFA probe to be positioned at a range of possible angles while still capturing the target neural structure, whereas conventional LRFA placement requires more fastidious, parallel positioning.^10^ These technical advantages may increase the probability of successful medial branch coagulation if variations or aberrancies in anatomy exist and provide a more reliable treatment effect in well-selected patients.

As such, the goal of the proposed study was to determine if individuals diagnosed with lumbar facet joint pain using dual-concordant MBBs with an ≥80% relief threshold demonstrate greater improvements in pain and function when treated with C-LRFA compared to FJI at both short and long-term follow-up. We hypothesized that treatment with C-LRFA would result in a greater likelihood of clinically meaningful improvements in pain and function compared to FJI.

## Methods

### Study design and patient selection

This IRB-approved (111315), single-blinded, randomized prospective comparative trial was conducted in a single tertiary academic center within the Department of Physical Medicine and Rehabilitation. Participants were recruited and treated between November 2018 and September 2021. All participants provided written informed consent before undergoing any active treatment interventions. Included were English-speaking adult patients aged ≥21 years, who had (1) unilateral or bilateral axial (non-radicular) low back pain for ≥3 months that had not responded to conservative treatment (physical therapy, oral analgesic agents, and non-invasive adjunctive treatments), (2) 7-day average numeric pain rating score (NPRS) for back pain of 5/10 or greater at baseline evaluation, and (3) had confirmed lumbar facet mediated pain (defined by ≥80% reduction of index pain after dual comparative diagnostic MBBs using 0.5 mL of 0.5% bupivacaine and 4% lidocaine, on respective encounters on separate days, at each of the appropriate medial branches). The MBBs were performed according to Spine Intervention Society guidelines.^9^ To qualify as a positive block, the individual had to experience ≥80% index pain relief from baseline, lasting at ≥1 hour with lidocaine and ≥2 hours with bupivacaine. In addition, the response from the bupivacaine had to have lasted longer than the lidocaine response. The exclusion criteria included (1) focal neurologic signs or symptoms, (2) lumbar radicular pain, (3) active systemic or local infections at the site of proposed needle and electrode placement, (4) coagulopathy or other bleeding disorder, (5) receipt of remuneration for their pain treatment (e.g., disability, worker's compensation, auto injury in litigation or pending litigation), (5) history of any lumbar or lower thoracic fusion surgery or placement of other hardware, (6) ≥grade two spondylolisthesis at an affected or adjacent level, (7) cobb angle >10 degrees, (8) sagittal vertical axis >5 degrees, (9) BMI >40, (10) incarceration, (11) allergy to local anesthetics, (12) widespread chronic pain or somatoform disorder (e.g., fibromyalgia), (13) prior LRFA, (14) addiction behavior, (15) severe clinical depression, anxiety, or any mental health condition with psychotic features, (16) possible pregnancy or other reason that precludes the use of fluoroscopy, and (17) daily chronic opiate use of >50 morphine equivalents.

### Randomization, masking, and crossover

A computer-generated 1:1 block randomization scheme (https://www.randomizer.org) was used to assign participants to receive RFA or FJI. Participants remained in their allocated groups until they met the crossover treatment criteria (<50% pain reduction after the index treatment at the 3-month follow-up), at which time, they were offered treatment crossover if they met these criteria. Participants could not be blinded to their intervention because of the significant procedural differences; however, the assessors were blinded.

### Data collection

Baseline demographics and other potential confounders were collected approximately four weeks before the index treatment. Baseline variables included age, sex, BMI, duration of low back pain, laterality of pain (ie, bilateral, symmetrical, unilateral), level and the number of painful facet joints, opioid use (binary yes/no), and NPRS back pain (7-day average). Validated patient-reported outcomes (PRO) were collected at baseline by assessors and again at the established post-procedural time points to compare pre- and post-PRO scores. In addition to the NPRS scores, Oswestery Disability Index (ODI) scores and Patient Global Impression of Change (PGIC) scores were collected. A blinded authorized study team member recorded all variables.

### Procedures

All C-LRFA and FJI procedures were performed under fluoroscopic guidance by experienced interventionalists with fellowship training in Pain Medicine or Interventional Spine Medicine. Patients were provided the option of moderate sedation using intravenous midazolam and/or fentanyl on a case-by-case basis.

#### C-LRFA procedure

The medial branches and the L5 dorsal ramus (when appropriate) were coagulated using an 18 gauge internally water-cooled RFA electrode (Coolief^®^ Cooled Radiofrequency Kit; Avanos Medical Inc, Alpharetta, GA). An identical technique was used to that previously described^28^ including C-RFA lesions performed for 165 seconds, with the RFA generator temperature set to 60°C (intralesional temperature >80 degrees).^31^

For bilateral low back pain, a maximum of four facet joints (two on each side) were denervated by C-RFA. For unilateral low back pain, a maximum of three facet joints were denervated by C-RFA.

#### FJI procedure

Lumbar facet joints were injected using a 22 or 25-gauge, 3.5 or 5-inch spinal needle (depending on body habitus). Needles were advanced under fluoroscopic guidance to enter the posterior facet joint space. The final needle position was confirmed in anterior-posterior and oblique views. Next, the stylet was removed, and extension tubing was attached. Approximately 0.2 mL of contrast media was injected to confirm intra-articular placement and the absence of vascular uptake. The injection was completed with 0.5 mL of 40 mg/mL Kenalog and 0.5 mL of 2% preservative-free lidocaine per facet joint.

A maximum of 4 facet joints (two on each side) were injected for bilateral low back pain. For unilateral low back pain, up to 3 facet joints were injected.

### Outcomes

The primary outcome for this study was the proportion of participants within each group (C-RFA and FJI) who experienced ≥50% reduction in NPRS score at 3 months. A ≥ 50% pain reduction is a common and recommended categorical outcome threshold that reflects a more significant pain reduction than the often reported minimal clinical import difference (MCID^32^) Secondary outcomes included between group differences in the proportion of participants in each group with 1) ≥30% decrease in ODI score (MCID^33^) 2) ≥6 score on the PGIC (“very much improved” or “much improved”), 3) ≥2-point improvement in NPRS score (MCID^32^) Secondary outcomes also included within and between group differences in NPRS, ODI, and PGIC continuous data. Serious adverse events were recorded.

### Power analysis

A power analysis was performed based on the previous observations of a single-arm cohort of 15 participants treated with conventional RFA^34^ and a separate study of 42 participants treated with FJI.^21^ Of the 15 participants treated with RFA, 87% experienced ≥60% reduction in pain at 10-month follow-up. Approximately 50% of the 42 participants treated with FJI reported treatment success at the 3-month follow-up. For the present power analysis, we used a conservative success rate (≥50% pain relief at 3 months) of 80% in the C-RFA group and 50% in the FJI group. Given that the planned primary outcome was categorical, with an alpha level of 0.05 and a power of 0.80, 39 participants in each group (a total of 78 participants) were found to be needed to detect a significant difference in the proportions between the two groups (80% vs 50%) using a χ^2^ test. To improve the power of the study beyond the minimally acceptable power standard and to account for an anticipated 20% attrition rate, we intended to enroll 120 participants in the trial.

### Data analysis

Descriptive statistics were calculated for participant demographics and clinical characteristics, along with outcome variables (7-day average NPRS and ODI scores) at baseline. Comparisons between treatment groups (C-LRFA vs FJI) were performed using Fisher exact tests for categorical variables and Wilcoxon-Mann-Whitney rank-sum tests for continuous variables.

Treatment success rates and corresponding 95% confidence intervals (CIs) were calculated for all outcome measures (≥50% NPRS reduction, ≥2-point reduction in NPRS, ≥50% reduction in OID, and ≥6 on the PGIC). Dichotomous variables representing the proportion of responders for each outcome measure were compared between C-LRFA vs FJI treatment groups at all follow-up time points using contingency table analyses with *χ*^2^ tests (or Fisher’s exact test when expected frequency values were low). Proportion ratios (PRs), along with 95% CIs, were calculated to quantify the size of the difference in proportions. Additionally, Wilcoxon-Mann-Whitney rank-sum tests were used to compare raw scores (continuous scale) for outcome measures by treatment at each follow-up time point. Participants who elected to cross over prior to 12 months and participants with missing data were considered treatment failures at all subsequent follow-up time points. If a participant elected to have their index procedure repeated, this was allowed per usual care standards and within the confines of insurance requirements (including at least 3 months and 6 months of >50% pain reduction following the index treatment, for FJI and C-LRFA, respectively), and not counted as a treatment failure.

Mixed-effects binomial logistic regression models^35^^,^^36^ with calculations of odds ratios (ORs) and 95% CIs were created to explore the relationships between outcome variables and selected covariates. The covariates of interest were treatment (C-LRFA vs FJI), follow-up time (1, 3, 6, and 12 months), and repeat of the index procedure. Furthermore, predicted probabilities of the outcome variable from each model were calculated to aid in the interpretations. All statistical analyses were performed using Stata/MP 17.0 (StataCorp, LLC, College Station, TX), with an *α* level of 0.05.

## Results

**Figure 1.**
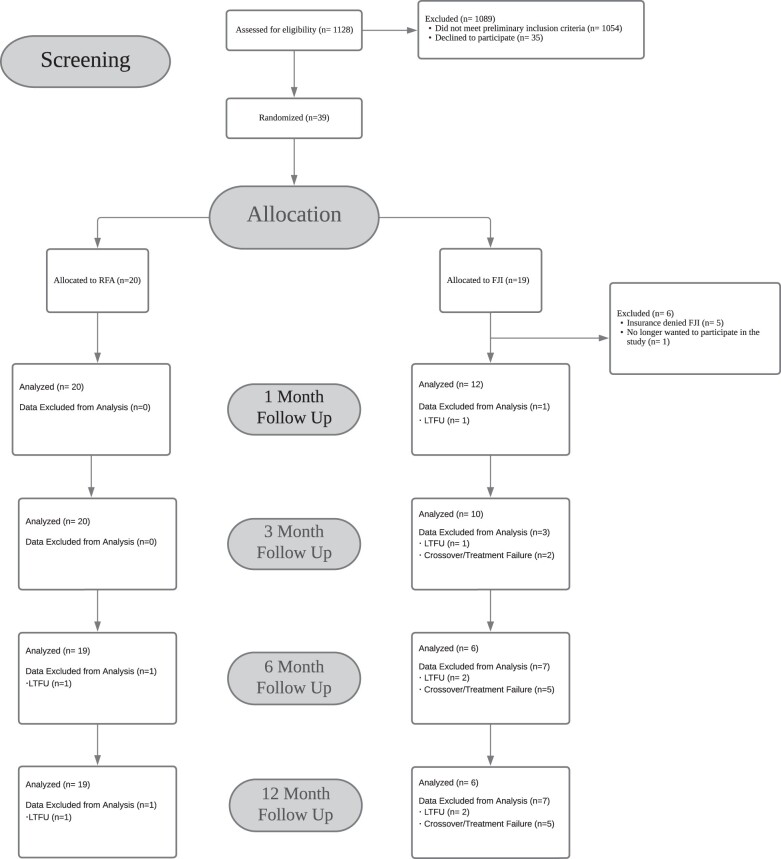
Consort diagram of participant flow through the study. FJI = facet joint steroid injection; LTFU = lost to follow-up; RFA = radiofrequency ablation.

One thousand one hundred twenty-eight patients with low back pain were assessed for eligibility. One thousand eighty-nine did not meet eligibility criteria and of those eligible, 35 declined participation. Thirty-nine patients were randomized, but 6 withdrew from the FJI group prior to study treatment. Five of these cases were due to insurance denial of FJI (the study treatment allocation), as most payers stopped coverage for FJI during the study recruitment period, and in one case the individual no longer wanted to participate in the study. Of the 13 remaining patients allocated to the FJI group, 1 individual was lost to follow-up immediately after receiving FJI treatment. Consequently, a total of 32 patients were analyzed in this study. Twenty and 12 patients received C-LRFA and FJI, respectively, as original treatments at the beginning of the study (Figure 1). Of note, enrollment was ended before goal recruitment was achieved because of the coverage change for FJI during the study perioid. The coverage changes resulted in study drop-out if a participant was randomized to FJI because this study utilized insurance-based pathways. When it became clear that 1:1 randomization was no longer possible, the study was ended early.

Participant demographics and clinical characteristics, along with outcome variables at baseline (NPRS and ODI), are summarized in Table 1. None of the demographic variables differed significantly between treatment groups (*P *>* *.05). Likewise, there was no significant between-group difference in baseline 7-day average NPRS or ODI, despite the trend that the FJI group showed higher scores than the RFA group for both variables (7-day average NPRS: 6.2 ± 2.2 vs 4.9 ± 1.4, *P *=* *.086; ODI: 19.5 ± 8.6 vs 15.1 ± 5.0, *P *=* *.056). The impact of these differences on the analysis was likely minimal since all outcome variables were change scores (dichotomous or continuous scale) from baseline to each follow-up time point, taking into account the between-participant differences in the baseline scores. Before the 12-month follow-up, 5 out of 12 participants (cross-over rate = 41.7%) who originally received FJI crossed over to C-LRFA, whereas zero patients (cross-over rate = 0.0%) in the C-LRFA group crossed over to FJI (*P *=* *.004).

**Table 1.** Demographics and clinical characteristics of patients (*N *=* *32).

	Original treatment		
Variable	FJI (*n *=* *12)	C-LRFA (*n *=* *20)	*P*
Gender			.358
Male	4 (33.3)	10 (50.0)	
Female	8 (66.7)	10 (50.0)	
Crossover before 12-month follow-up			**.004**
Yes	5 (41.7)^b^	0 (0.0)	
No	7 (58.3)	20 (100.0)	
Laterality			.267
Unilateral	3 (25.0)	10 (50.0)	
Bilateral	9 (75.0)	10 (50.0)	
Opioid use at baseline			.540
Yes	2 (16.7)	1 (5.0)	
No	10 (83.3)	19 (95.0)	
Age (yr) [mean (SD)]	62.4 (14.6)	62.2 (12.4)	.694^a^
Body mass index (kg/m^2^) [mean (SD)]	25.7 (5.3)	28.7 (5.3)	.136^a^
Chronicity (yr) [mean (SD)]	5.4 (0.9)	4.9 (1.1)	.156^a^
Total number of joints denervated [mean (SD)]	1.9 (0.3)	2.1 (0.3)	.307^a^
7-day average NPRS at baseline [mean (SD)]	6.2 (2.2)	4.9 (1.4)	.086^a^
ODI at baseline [mean (SD)]	19.5 (8.6)	15.1 (5.0)	.056^a^

Values are frequency (%), and *P* values are from Fisher exact test unless specified otherwise.

C-LRFA = Cooled Lumbar Radiofrequency Ablation; FJI = Facet Joint Injection; NPRS = Numerical Pain Rating Scale score; ODI = Oswestry Disability Index score.

a From Wilcoxon-Mann-Whitney rank-sum test.

b At 1 month (*n *=* *1), 3 month (*n *=* *1), 4 month (*n *=* *1), and 5 month (*n *=* *2).

**Figure 2.**
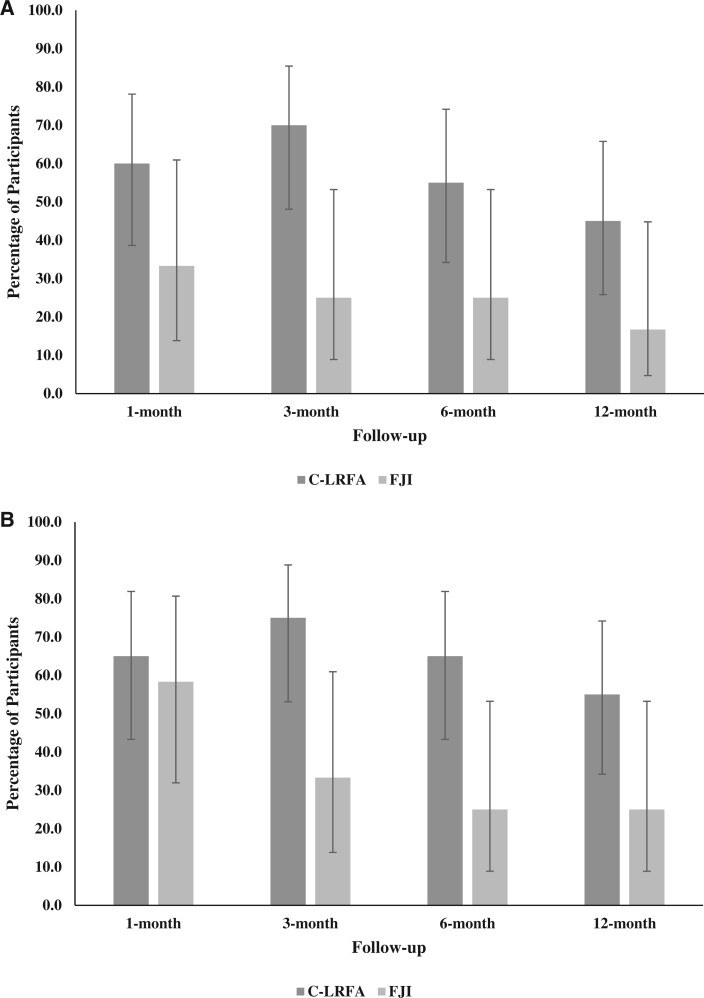
**(A)** Proportion of participants who experienced ≥ 50% reduction in 7-day average NRS by treatment at follow-up time points. Whiskers represent 95% confidence intervals. **(B)** Proportion of participants who experienced ≥ 2-point reduction in 7-day average NRS by treatment at follow-up time points. Whiskers represent 95% confidence intervals.

Outcomes for 7-day average NPRS at 1, 3, 6, and 12 months post-treatment for both groups are shown in Table 2 and Figure 2. At 3-month follow-up, participants who received C-LRFA showed significantly higher rates of treatment success compared to those who received FJI, as measured by both ≥50% NPRS reduction (the primary outcome; *P *=* *.014) and ≥2-point NPRS reduction (*P *=* *.030). Specifically, 70.0% (*n *=* *14) and 75.0% (*n *=* *15) of the 20 participants in the C-LRFA group showed ≥50% NPRS reduction and ≥2-point NPRS reduction, respectively, compared with 25.0% (*n *=* *3) and 33.3% (*n *=* *4) of the 12 participants in the FJI group. PRs for ≥50% NPRS reduction and ≥2-point NPRS reduction were 2.80 (95% CI = 1.01, 7.77) and 2.25 (0.97, 5.21), indicating that C-LRFA resulted in about 2.8 and 2.3 times greater reductions in these variables compared with FJI. Similarly, at 6-month follow-up, a significantly higher percentage of participants in the C-LRFA group showed ≥2-point NPRS reduction compared to the FJI group [65.0% (*n *=* *13) vs 25.0% (*n *=* *3), *P *=* *.028], with a PR of 2.60 (95% CI = 0.93, 7.29).

**Table 2.** 7-day average NPRS from baseline to follow-up time points by treatment.

		Original treatment			
Follow-up	Variable	FJI (*n *=* *12)	C-LRFA (*n *=* *20)	*P*	PR (95% CI)
**1 month**	≥ 50% NPRS reduction [n (%)]			.144	1.80 (0.75, 4.32)
	Yes	4 (33.3)	12 (60.0)		
	No	8 (66.7)	8 (40.0)		
	≥ 2-point NPRS reduction [n (%)]			.724^a^	1.11 (0.63, 1.98)
	Yes	7 (58.3)	13 (65.0)		
	No	5 (41.7)	7 (35.0)		
	Raw NPRS score [mean (SD)]				
	At 1 month	3.2 (1.7)	2.4 (2.1)	.306^b^	
	Change (baseline minus 1 month)	2.8 (2.6)	2.4 (2.1)	.739^b^	
**3 month**	≥ 50% NPRS reduction [n (%)]			**.014**	2.80 (1.01, 7.77)
	Yes	3 (25.0)	14 (70.0)		
	No	9 (75.0)	6 (30.0)		
	≥ 2-point NPRS reduction [n (%)]			**.030** ^a^	2.25 (0.97, 5.21)
	Yes	4 (33.3)	15 (75.0)		
	No	8 (66.7)	5 (25.0)		
	Raw NPRS score [mean (SD)]				
	At 3 month	4.1 (2.0)	2.3 (2.0)	**.010** ^b^	
	Change (baseline minus 3 month)	1.9 (2.7)	2.6 (2.2)	.276^b^	
**6 month**	≥ 50% NPRS reduction [n (%)]			.098	2.20 (0.76, 6.33)
	Yes	3 (25.0)	11 (55.0)		
	No	9 (75.0)	9 (45.0)		
	≥ 2-point NPRS reduction [n (%)]			**.028**	2.60 (0.93, 7.29)
	Yes	3 (25.0)	13 (65.0)		
	No	9 (75.0)	7 (35.0)		
	Raw NPRS score [mean (SD)]				
	At 6 month	4.1 (2.8)	2.5 (2.1)	.118^b^	
	Change (baseline minus 6 month)	2.0 (3.1)	2.5 (2.2)	.431^b^	
**12 month**	≥ 50% NPRS reduction [n (%)]			.139^a^	2.70 (0.70, 10.46)
	Yes	2 (16.7)	9 (45.0)		
	No	10 (83.3)	11 (55.0)		
	≥ 2-point NPRS reduction [n (%)]			.098	2.20 (0.76, 6.33)
	Yes	3 (25.0)	11 (55.0)		
	No	9 (75.0)	9 (45.0)		
	Raw NPRS score [mean (SD)]				
	At 12 month	4.8 (2.7)	2.9 (2.4)	.133^b^	
	Change (baseline minus 12 month)	1.3 (3.2)	1.9 (2.9)	.675^b^	

*P* values are from *χ*^2^ test, unless specified otherwise.

CI = Confidence Interval; C-LRFA = Cooled Lumbar Radiofrequency Ablation; FJI = Facet Joint Injection; NPRS = Numerical Pain Rating Scale score; PR = Proportion Ratio; SD = Standard Deviation.

a From Fisher exact test.

b From Wilcoxon-Mann-Whitney rank-sum test.

**Figure 3.**
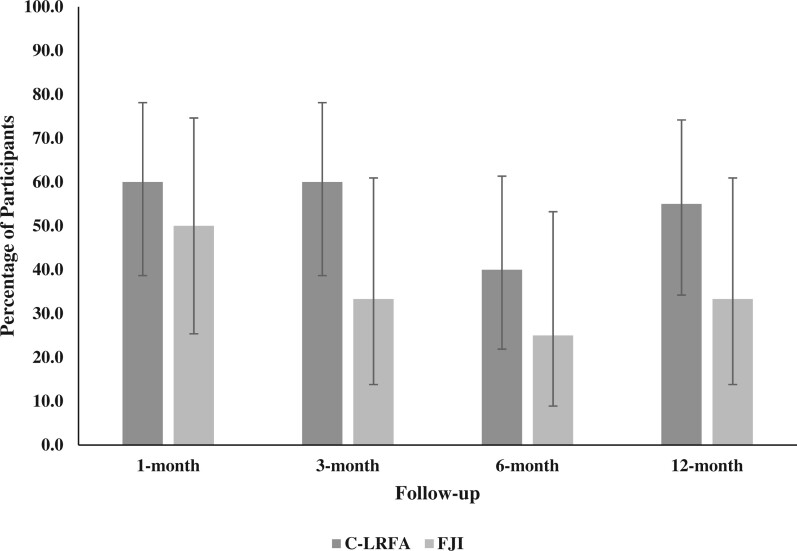
Proportion of participants who experienced ≥ 30% reduction in ODI score by treatment at follow-up time points. Whiskers represent 95% confidence intervals.

Improvements in function as measured by ODI did not significantly differ between treatment groups (Table 3). There was no significant difference in ≥ 15-point ODI reduction or ≥30% ODI reduction between the C-LRFA and FJI groups at any follow-up time point (*P *>* *.05). More participants reported treatment success at all follow-up time points as measured by ≥30% ODI reduction when comparing C-LRFA vs FJI, but this difference did not reach statistical significance (Figure 3).

**Table 3.** ODI from baseline to follow-up time points by treatment.

		Original treatment			
Follow-up	Variable	FJI (*n *=* *12)	C-LRFA (*n *=* *20)	*P*	PR (95% CI)
**1 month**	≥ 15-point ODI reduction [n (%)]			.999^a^	1.80 (0.21, 15.41)
	Yes	1 (8.3)	3 (15.0)		
	No	11 (91.7)	17 (85.0)		
	≥ 30% ODI reduction [n (%)]			.581	1.20 (0.61, 2.34)
	Yes	6 (50.0)	12 (60.0)		
	No	6 (50.0)	8 (40.0)		
	Raw ODI score [mean (SD)]				
	At 1 month	11.6 (5.5)	7.9 (5.6)	.116^b^	
	Change (baseline minus 1 month)	6.3 (7.5)	6.6 (6.4)	.973^b^	
**3month**	≥ 15-point ODI reduction [n (%)]			.999^a^	1.80 (0.21, 15.41)
	Yes	1 (8.3)	3 (15.0)		
	No	11 (91.7)	17 (85.0)		
	≥ 30% ODI reduction [n (%)]			.144	1.80 (0.75, 4.32)
	Yes	4 (33.3)	12 (60.0)		
	No	8 (66.7)	8 (40.0)		
	Raw ODI score [mean (SD)]				
	At 3 month	11.7 (5.3)	8.4 (6.1)	.201^b^	
	Change (baseline minus 3 month)	6.8 (7.1)	6.7 (6.2)	.923^b^	
**6 month**	≥ 15-point ODI reduction [n (%)]			.999^a^	1.20 (0.26, 5.59)
	Yes	2 (16.7)	4 (20.0)		
	No	10 (83.3)	16 (80.0)		
	≥ 30% ODI reduction [n (%)]			.465^a^	1.60 (0.52, 4.89)
	Yes	3 (25.0)	8 (40.0)		
	No	9 (75.0)	12 (60.0)		
	Raw ODI score [mean (SD)]				
	At 6 month	10.6 (7.1)	8.8 (7.1)	.605^b^	
	Change (baseline minus 6 month)	8.0 (9.0)	5.8 (7.5)	.515^b^	
**12 month**	≥ 15-point ODI reduction [n (%)]			.338^a^	0.40 (0.08, 2.06)
	Yes	3 (25.0)	2 (10.0)		
	No	9 (75.0)	18 (90.0)		
	≥ 30% ODI reduction [n (%)]			.234	1.65 (0.68, 4.03)
	Yes	4 (33.3)	11 (55.0)		
	No	8 (66.7)	9 (45.0)		
	Raw ODI score [mean (SD)]				
	At 12 month	12.4 (6.0)	8.8 (6.6)	.374^b^	
	Change (baseline minus 12 month)	5.6 (9.4)	6.1 (6.6)	.692^b^	

*P* values are from *χ*^2^ test, unless specified otherwise.

CI = Confidence Interval; C-LRFA = Cooled Lumbar Radiofrequency Ablation; FJI = Facet Joint Injection; ODI = Oswestry Disability Index score; PR = Proportion Ratio; SD = Standard Deviation.

a From Fisher exact test.

b From Wilcoxon-Mann-Whitney rank-sum test.

**Figure 4.**
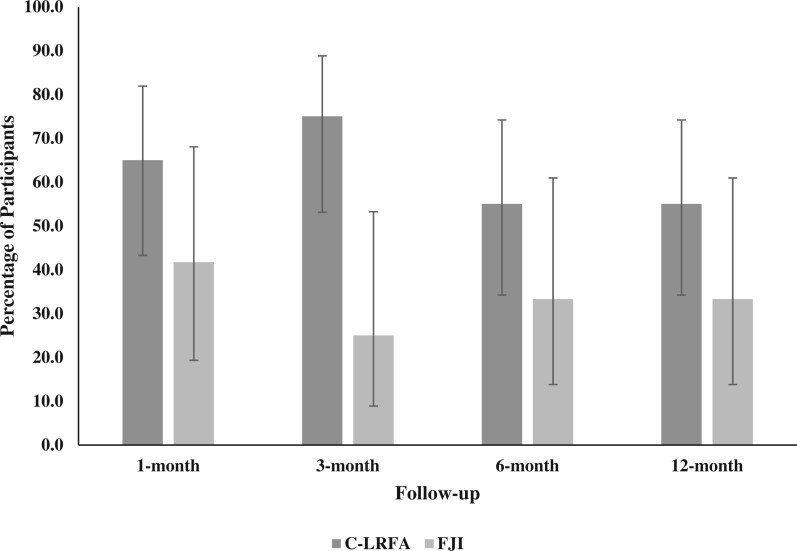
Proportion of participants who experienced ≥ 6 in PGIC by treatment at follow-up time points. Whiskers represent 95% confidence intervals.

PGIC scores at follow-up time points are summarized in Table 4 and Figure 4. A significantly larger proportion of participants in the C-LRFA group reported ≥6 in PGIC at 3 months compared with those in the FJI group [75.0% (*n *=* *15) vs 25.0% (*n *=* *3), *P *=* *.006]. Specifically, C-LRFA (vs FJI) resulted in ≥6 in PGIC by a three-fold magnitude (PR = 3.00, 95% CI = 1.09, 8.25). Furthermore, raw PGIC scores were significantly higher in the C-LRFA group than in the FJI group at 3-month follow-up (*P *=* *.013). Similar to the ODI outcomes, a larger proportion of participants in the C-LRFA compared to FJI group reported treatment success defined by PGIC score ≥6, but this did not reach statistical significance at the 6- and 12-month time points (*P *>* *.05).

**Table 4.** PGIC at follow-up time points by treatment.

		Original treatment			
Follow-up	Variable	FJI (*n *=* *12)	C-LRFA (*n *=* *20)	*P*	PR (95% CI)
**1 month**	≥ 6 in PGIC [n (%)]			.198	1.56 (0.74, 3.28)
	Yes	5 (41.7)	13 (65.0)		
	No	7 (58.3)	7 (35.0)		
	Raw PGIC score [median (IQR)]				
	At 1 month	5.5 (1)	6 (2)	.344^a^	
**3 month**	≥ 6 in PGIC [n (%)]			**.006**	3.00 (1.09, 8.25)
	Yes	3 (25.0)	15 (75.0)		
	No	9 (75.0)	5 (25.0)		
	Raw PGIC score [median (IQR)]				
	At 3 month	5 (1)	6 (1.5)	**.013** ^a^	
**6 month**	≥ 6 in PGIC [n (%)]			.234	1.65 (0.68, 4.03)
	Yes	4 (33.3)	11 (55.0)		
	No	8 (66.7)	9 (45.0)		
	Raw PGIC score [median (IQR)]				
	At 6 month	6 (1)	6 (3)	.700^a^	
**12 month**	≥ 6 in PGIC [n (%)]			.234	1.65 (0.68, 4.03)
	Yes	4 (33.3)	11 (55.0)		
	No	8 (66.7)	9 (45.0)		
	Raw PGIC score [median (IQR)]				
	At 12 month	5.5 (4)	6 (2)	.141^a^	

*P* values are from *χ*^2^ test, unless specified otherwise.

C-LRFA = Cooled Lumbar Radiofrequency Ablation; FJI = Facet Joint Injection; IQR = Interquartile Range; PGIC = Patient Global Impression of Change; PR = Proportion Ratio.

a From Wilcoxon-Mann-Whitney rank-sum test.

**Figure 5.**
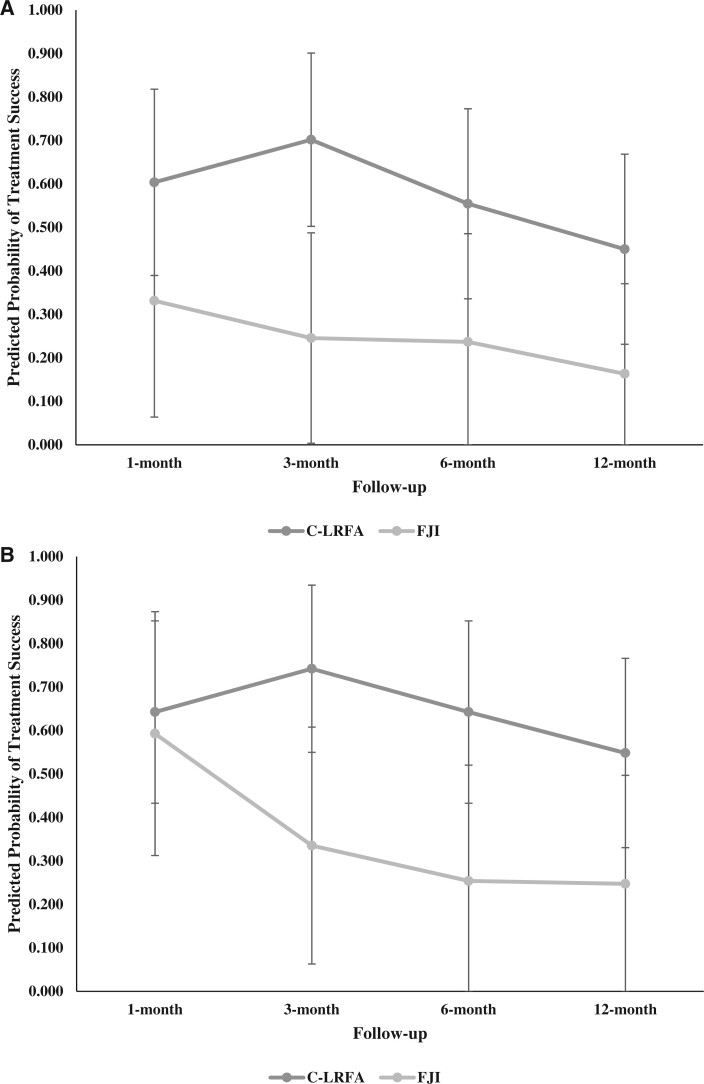

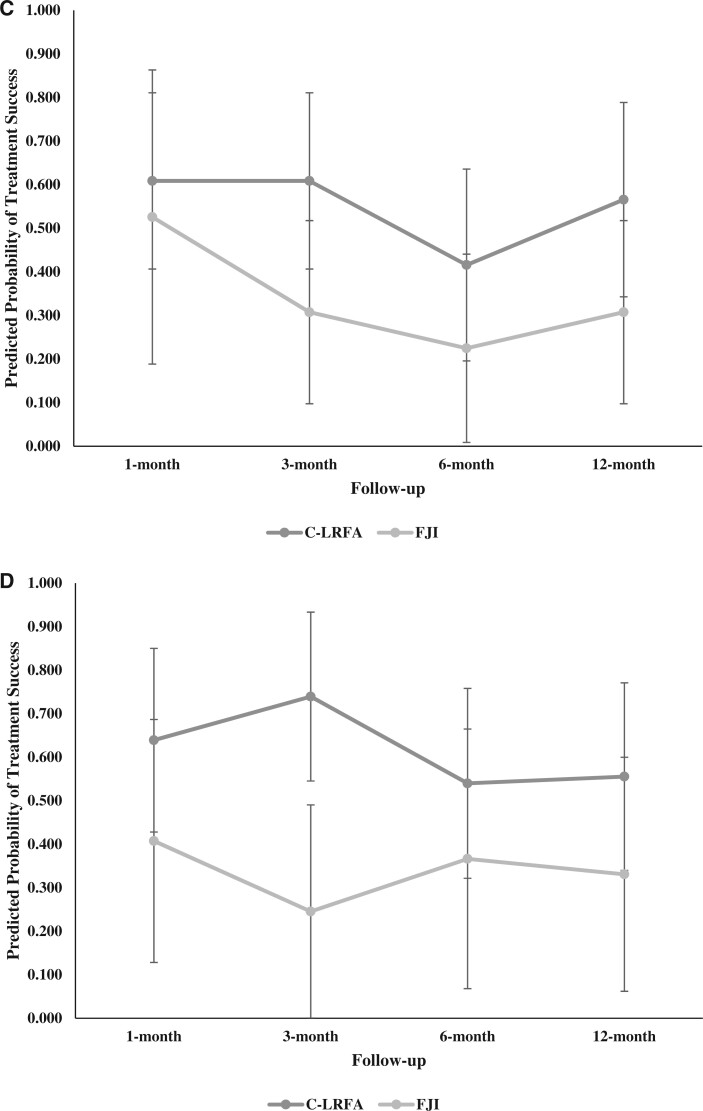
**(A)** Predicted probabilites of achieving ≥ 50% NRS reduction by treatment at follow-up time points. Whiskers represent 95% confidence intervals. **(B)** Predicted probabilites of achieving ≥ 2-point NRS reduction by treatment at follow-up time points. Whiskers represent 95% confidence intervals. **(C)** Predicted probabilites of achieving ≥ 30% ODI reduction by treatment at follow-up time points. Whiskers represent 95% confidence intervals. **(D)** Predicted probabilites of achieving ≥ 6 in PGIC by treatment at follow-up time points. Whiskers represent 95% confidence intervals.

Table 5 shows the results of the mixed-effects binomial logistic regression models for NPRS, ODI, and PGIC outcomes. The main effect of original treatment was significant within the models with regard to ≥50% NPRS reduction, ≥2-point NPRS reduction, and ≥6 in PGIC (*P *<* *.05). The odds of achieving ≥50% NPRS reduction for C-LRFA was more than seven times the odds for FJI (OR = 7.49; 95% CI = 1.60, 35.01; *P *=* *.010). On average, the predicted probabilities of achieving ≥50% NPRS reduction for C-LRFA and FJI during the study were 0.577 (95% CI = 0.434, 0.721) and 0.244 (95% CI = 0.087, 0.401), respectively. The predicted probabilities throughout the follow-up time points were consistently higher in the C-LRFA group than in the FJI group (Figure 5A). Similarly, the odds of achieving ≥2-point NPRS reduction for C-LRFA was over five times greater than the odds for FJI (OR = 5.28; 95% CI = 1.20, 23.14; *P *=* *.027). The overall predicted probabilities of achieving ≥2-point NPRS reduction for C-LRFA and FJI were 0.643 (95% CI = 0.503, 0.783) and 0.362 (95% CI = 0.179, 0.546), respectively, with the trend of probabilities higher in the C-LRFA group than in the FJI group throughout the follow-up time points (Figure 5B). Further, a greater treatment effect of C-LRFA compared with FJI was shown by ≥6 in PGIC (OR = 4.30; 95% CI = 1.29, 14.34; *P *=* *.018). The overall predicted probabilities of achieving ≥6 in PGIC for C-LRFA and FJI during the study were 0.619 (95% CI = 0.487, 0.751) and 0.336 (95% CI = 0.169, 0.503), respectively (Figure 5C).

**Table 5.** Mixed-effects binomial logistic regression models on treatment effects by FJI and C-LRFA.

Outcome	Covariate	OR	95% CI	*P*
≥ 50% NPRS reduction	Original treatment (vs FJI)			
	C-LRFA	7.49	1.60, 35.01	**.010**
	Follow-up time (vs 1 month)			
	3 month	1.20	0.36, 4.04	.765
	6 month	0.67	0.20, 2.29	.522
	12 month	0.37	0.10, 1.34	.129
	Repeat index procedure (vs no)			
	Yes	1.27	0.15, 10.82	.829
≥ 2-point NPRS reduction	Original treatment (vs FJI)			
	C-LRFA	5.28	1.20, 23.14	**.027**
	Follow-up time (vs 1 month)			
	3 month	0.83	0.24, 2.88	.768
	6 month	0.49	0.14, 1.75	.270
	12 month	0.33	0.09, 1.20	.092
	Repeat index procedure (vs no)			
	Yes	0.58	0.07, 4.64	.605
≥ 15-point ODI reduction	*Convergence not achieved*			
≥ 30% ODI reduction	Original treatment (vs FJI)			
	C-LRFA	6.92	0.46, 104.10	.162
	Follow-up time (vs 1 month)			
	3 month	0.56	0.12, 2.55	.455
	6 month	0.13	0.02, 0.71	**.019**
	12 month	0.43	0.09, 2.10	.299
	Repeat index procedure (vs no)			
	Yes	0.73	0.04, 13.89	.837
≥ 6 in PGIC	Original treatment (vs FJI)			
	C-LRFA	4.30	1.29, 14.34	**.018**
	Follow-up time (vs 1 month)			
	3 month	1.02	0.32, 3.19	.979
	6 month	0.64	0.20, 2.06	.460
	12 month	0.64	0.20, 2.03	.449
	Repeat index procedure (vs no)			
	Yes	0.51	0.06, 4.10	.528

CI = Confidence Interval; C-LRFA = Cooled Lumbar Radiofrequency Ablation; FJI = Facet Joint Injection; NPRS = Numerical Pain Rating Scale score; ODI = Oswestry Disability Index score; OR = Odds Ratio; PGIC = Patient Global Impression of Change.

### Adverse events

There were no serious adverse events reported by any participant within either group.

## Discussion

We report outcomes of the first pragmatic randomized trial comparing C-LRFA to FJI for treating lumbar facet pain in patients diagnosed with lumbar facet joint pain using the clinical criterion of ≥80% pain reduction in reponse to dual-comparative MBBs.^20–22^ In this study, participants treated with C-LRFA were ∼7 times more likely to have ≥50% pain reduction (OR 7.49 [95% CI 1.60, 35.01]) and were ∼4 times more likely to achieve MCID on PGIC (OR 4.30 [95% CI 1.29, 14.34]) than those treated with FJI. The elevated OR results from a significantly higher responder rate in the L-CRFA group compared to the FJI group.

Patient selection in both research and clinical care is vital and likely contributed to the observed outcomes in the present study. In addition to excluding patients with conditions known to reduce treatment response (ie, widespread pain or somatoform disorders, concomitant lumbar pain disorders, depression, etc.), patients had to experience a positive response to dual comparative MBBs with pain reduction of ≥80% of concordant duration to that expected of two different local anesthetics used on independent occasions.^10^^,^^11^ A systematic review^14^ and multi-society consensus guidelines^15^ agree that LRFA outcomes are improved with more strict medial branch block criteria (increasing the number of blocks and required pain reduction after each block) and reduced with more relaxed standards. The current study implemented block criteria less strictly than some LRFA studies that have required 100% pain relief after dual MBBs, but more stringent than the consensus guideline recommendation of 50% pain relief after a single block.^15^ The block criteria in the present study were designed to reflect the recommendation by the Spine Intervention Society and what is required by the Centers for Medicare and Medicaid Services.^37^ The responder rate in this study (50% achieving ≥50% pain relief at 6 months) is lower than pooled RFA outcome (60% reaching 100% pain relief at 6 months) for individuals who experience 100% pain relief with dual comparative MBBs^14^ but is higher than the responder rate (39% achieving ≥50% at 6 months^38^) in those selected by ≥50% pain reduction after a single block.

The C-LRFA electrode lesion size and forward-projecting, spherical geometry may have contributed to the positive outcomes observed in this study. C-LRFA electrodes produce a more significant lesion by using an internal perfusate that serves as a heat sink in tissue adjacent to the electrode. Electrode cooling prevents adjacent tissue charing, lowering tissue impedance, and allowing the lesion to develop further from the electrode tip in a spherical shape. The C-LRFA lesion shape and size enable proceduralists to approach the lumbar medial branches from a variety of angles without sacrificing target neural capture, unlike the traditional parallel L-RFA technique. Cendeno et al.^39^ compared conventional monopolar and cooled electrode lesion volume and shapes in 10 chicken breast specimens. They found that the cooled electrodes (identical to the electrodes used in the current study) produced the most extensive mean lesion volume (595 mm^3^) followed by the 16 g monopolar electrode (360 mm^3^). The current study results, along with prior studies,^34^^,^^40^ provide direct and indirect evidence that larger ablation zones/volumes may lead to better outcomes. It is unclear if the therapeutic benefit of C-LRFA results from more considerable neural segmental destruction and/or if it increases the maximal tolerable margin of technical error and helps reduce the impact of anatomical nerve variation.

Interestingly, there was no significant difference in C-LRFA and FJI responder rates as assessed by ≥15-point or ≥30% ODI reduction. The lack of a between-group difference suggests that C-LRFA does not reduce ODI scores to a greater degree than FJI; however, these findings are more likely due to a small sample size and the lack of a minimum ODI score as a study eligibility criterion (which creates a “floor”-effect). There was a trend that C-LRFA was ∼7 times more likely to result in ≥30% ODI reduction compared to FJI, but this trend did not reach statistical significance (OR 6.92 [95% CI 0.46, 104.10]). Of those participants treated with L-CRFA, 60%, 40%, and 55% achieved ≥30% ODI reduction at 3,6 and 12 months. These rates are similar to the 60% responder rate observed in the only other C-LRFA trial using ODI as a disability outcome measure.^28^ It should be noted that all L-RFA studies^34^^,^^40^^,^^41^ using the ≥80% pain reduction after dual MBB have found significant functional improvement; however, none of these studies measured ODI scores.

This study has limitations, including a lack of participant blinding and small sample size. Blinding participants in this study was not feasible for financial reasons. During study initiation, FJI and C-LRFA were both covered by insurance but had different respective costs. If a patient was blinded, insurance could not be appropriately billed, which would have made the present study prohibitively expensive and not feasible. The absence of participant blinding and issues with obtaining insurance authorization for FJI may partially explain why no participants crossed over to FJI after their index C-LRFA procedure. Non-blinded participants allocated to the C-LRFA study arm may have been less interested in steroid injection if they believed they had already received the “superior” treatment, while insurance denial for FJI could have deterred individuals who were otherwise inclined to pursue this treatment option. The sample size is a significant issue. As reported in our power calculation, we planned to enroll 120 patients (60 in each group). Enrollment was ended before goal recruitment was achieved because of the coverage change for FJI during the study period. It became clear that 1:1 randomization was no longer possible, so the study was ended early. Finally, a large number of patients were screened (all-comers with CLBP) in order to enroll a final total of 32. We used strict inclusion criteria to identify patients with “pure” or highly dominant facet joint pain without significant concomitant pain generators. As such, generalizability of the present findings is limited to individuals with dominant facet joint pain.

## Conclusion

We report outcomes for the first pragmatic randomized trial comparing C-LRFA to FJI for treating lumbar facet pain in patients diagnosed with lumbar facet joint pain using the clinical criterion of ≥80% pain reduction in reponse to dual-comparative MBBs. In this study, C-LRFA demonstrated superior success rates compared to FJI across pain and functional outcome domains at the primary endpoint. In regression modeling, the odds of achieving ≥50% NPRS reduction for C-LRFA was more than 7 times the odds for FJI. This study reinforces the previous finding that lumbar radiofrequency ablation is an effective treatment for appropriately selected patients with lumbar facet joint pain.
